# Minimally Invasive Screw Fixation of Unstable Pelvic Fractures Using the “Blunt End” Kirschner Wire Technique Assisted by 3D Printed External Template

**DOI:** 10.1155/2019/1524908

**Published:** 2019-10-24

**Authors:** Kaifang Chen, Sheng Yao, Fan Yang, Deepak Drepaul, Dionne Telemacque, Fengzhao Zhu, Lian Zeng, Zekang Xiong, Tingfang Sun, Xiaodong Guo

**Affiliations:** Department of Orthopaedics, Union Hospital, Tongji Medical College, Huazhong University of Science & Technology, Jiefang Avenue No. 1277, Wuhan, Hubei 430022, China

## Abstract

**Objective:**

This study aimed to determine the accuracy and safety of the “blunt end” Kirschner wire (KW) technique for the minimally invasive treatment of unstable pelvic fractures with the assistance of a 3D printed external template.

**Methods:**

Clinical data of 28 patients with unstable pelvic fractures between January 2016 and January 2018 were retrospectively analyzed. There were 6 cases of B1, 10 of B2, 8 of C1, and 4 of C2 fractures, all of which received surgical treatment. The “blunt end” KW technique with a 3D template was adopted for the minimally invasive placement of the iliosacral (IS) or superior ramus screws. The number of intraoperative fluoroscopies, surgical time, and complications were recorded. Postoperative reduction was assessed using the Matta criteria, and the Majeed score system was used to evaluate postoperative functional recovery.

**Results:**

The average number of fluoroscopies was 35 per patient, and the average surgical time was 85.2 min. A total of 19 S1 and 28 S2 IS screws were inserted. Eleven antegrade superior ramus screws and 4 retrograde screws were placed in 11 patients, and anterior subcutaneous internal fixation (INFIX) was used to fix the anterior pelvic ring in 17 patients. All patients were followed up for an average of 18 months. Postoperative reduction was evaluated by Matta's criteria: excellent in 16 cases, good in 9 cases, and fair in 3 cases. The Majeed score was used in the last follow-up to evaluate functional recovery: excellent in 13 cases, good in 10 cases, fair in 4 cases, and poor in 1 case. There were no cases of operative vascular injury.

**Conclusion:**

The “blunt end” KW technique with a 3D printed external template is a safe and effective method for the placement of IS and superior ramus screws in unstable pelvic fractures with minimized surgical duration and radiation exposure.

## 1. Introduction

Treatment of unstable pelvic fractures using closed reduction and internal fixation (CRIF), by virtue of its minimally invasion and stable fixation nature, has become more popular in recent years [1, 2]. The percutaneous corridor screw technique is the main fixation method of CRIF, including percutaneous iliosacral (IS), superior ramus (antegrade or retrograde), and LC-2 screws [3–5]. In particular, IS screw fixation is a known standard method for the management of unstable posterior pelvic ring injuries, providing adequate biomechanical stability [1, 6, 7]. The conventional percutaneous technique uses fluoroscopic guidance to complete freehand screw placement. However, due to a variety of factors including patients' obesity, congenital anomalies, or surgeons' inexperience, the procedure may result in screw malpositioning (2%–15%) and severe neurovascular injury [8, 9]. Moreover, the prolonged radiation exposure that accompanies fluoroscopic guidance also discouraged many orthopedic surgeons from performing the percutaneous technique.

The use of 3D image guidance and navigation technology enables more accurate percutaneous screw placement [10, 11]. However, the high cost and excessive radiation exposure have limited its widespread application. With the broad implementation of 3D printing technology in the medical field, it is a good alternative to 3D navigation technology to use the preoperative 3D computed tomography (CT) image of patients and reverse engineering technology to design personalized templates for pelvic corridor screw insertions. Currently, for example, the template for IS screw insertion was designed based on the specific anatomical features (iliospinale posterius), and a 4-5 cm incision on the iliospinale posterius (posterior iliac crest) was necessary and all attached muscle/soft tissue should be dissected during the operation [12]. This is called internal template, which is not actually minimally invasive. Moreover, it is difficult to find accurate anatomical features to match with the template intraoperatively. Therefore, the precision of screw insertions is questionable [13].

To minimize these drawbacks, we proposed an external template based on pelvic external fixation pins [13]. The design principle is similar to the internal template mentioned above. However, as an extension of the patient's pelvic structure, the pelvic external fixation pins (cylindrical) with a simple geometric surface provide a solid cornerstone for designing and matching well with the template. This design allows the template to be firmly and accurately attached to the external fixation pin without incising. At the same time, in terms of surgical techniques, we also made a small modification to the screw placement. A 2.5 mm KW has a sharp and a blunt end, conventionally the sharp end is advanced in an antegrade fashion into the medullary canal for the guidance of cannulated screw insertion. If the orientation of the KW is slightly deviated, the sharp tip can easily penetrate the bone cortex resulting in adverse outcomes. Taking this into consideration and inspired by the “antegrade-retrograde” KW technique proposed by Peled et al. [14], an idea of a possibly safer method came to mind: reversing the KW and using the blunt end to advance into the medullary corridor.

The purpose of the study was to describe our experience of minimally invasive screw fixation of unstable pelvic ring injuries using the “blunt end” KW technique assisted by 3D printed external template and to explore its feasibility, safety, and clinical effectiveness.

## 2. Materials and Methods

### 2.1. Inclusion and Exclusion Criteria

Inclusion criteria: (1) patients with unstable pelvic fracture (tile type B or C); (2) patients older than 18 years old; (3) patients with a closed pelvic fracture; (4) fracture with no or slight displacement on at least 1 side of the hemipelvic ring; (5) follow-up data complete and follow-up time >1 year.

Exclusion criteria: (1) open pelvic fracture; (2) those who have serious complications and cannot withstand anesthesia or surgery; (3) patients with severe ankylosing spondylitis involving the sacroiliac joint; (4) those who did not complete the follow-up and had incomplete imaging data.

### 2.2. General Information

Between January 2016 and January 2018, a series of 28 patients with unstable pelvic fractures were treated using the “antegrade-retrograde” KW technique with the assistance of 3D printed external template in our trauma center. These patients consisted of 19 males and 9 females with an average age of 46.4 years and an age range of 18–68 years. The fractures were classified according to the Tile classification [15], including 6 type B1, 10 B2, 8 C1, and 4 C2 fractures. The cause of injury was car accident in 16 cases and high-level fall in 12 cases. The time from injury to operation ranged from 7 to 32 days, with an average of 12 days. All patients in this study obtained and signed the informed consent.

### 2.3. Preoperative Design and Rapid-Prototype of External Guide Template

**Figure 1:**
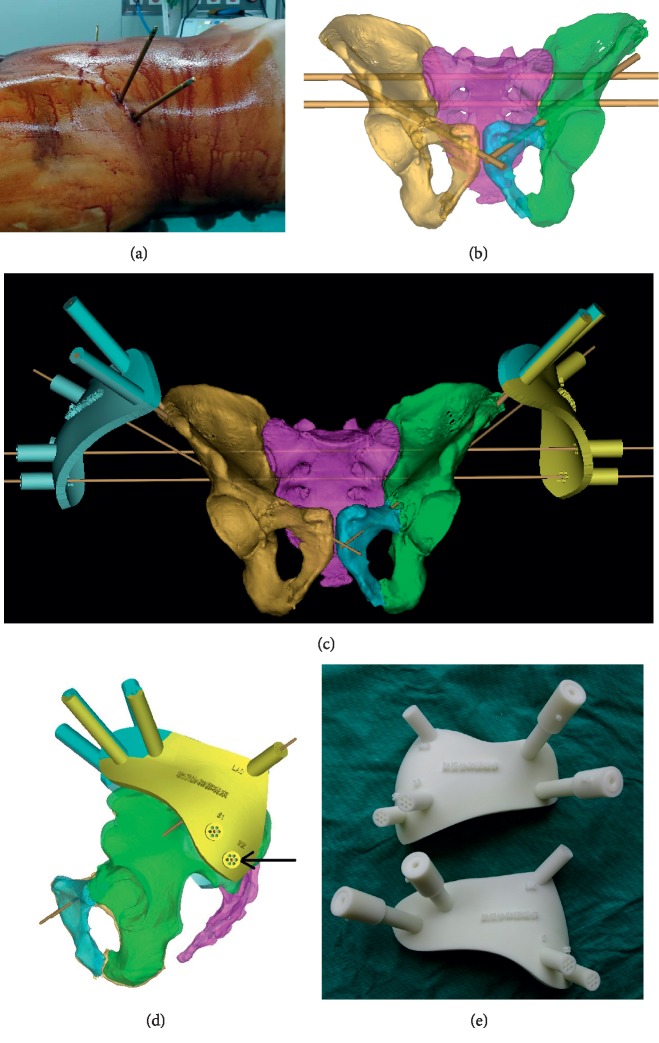
Photographs demonstrating the design and 3D printing of the external guide template. (a) Two nonparallel external fixation pins were inserted into the patient's iliac crest as markers for the design of the template. (b) 7.0 mm diameter cylinders were employed to simulate the tracks of S1, S2 iliosacral, or superior ramus screws. (c) The external template was designed based on the marker pins and virtual screw guide pins (2.5 mm diameter KW), and the template was cut into 2 pieces (shown in different colors) to facilitate the smooth sticking of the external fixation pins during the operation. (d) “Plum blossom” like guide holes (black arrow) could facilitate the microadjustment of the KW. (e) The 3D printed external template.

Emergent unilateral or bilateral external fixation pins were implanted in the iliac crest for each patient at the time of admission (Figure 1(a)). Patients were then asked to undergo CT scan of the pelvis. The radiologist was instructed to make sure the CT scan included the tail end of the external fixation pins on the iliac crest, in order to ensure the design accuracy of the external guide template. Subsequently, the patient's CT data were imported into the Mimics 10.01 software (Materialise, Belgium) in DICOM format for 3D modeling. Generally cannulated screws with a diameter of 6.5 mm were used for pelvic fracture fixation; therefore, we employed 7.0 mm diameter cylinders to simulate the tracks of S1, S2 IS, or superior ramus screws (Figure 1(b)). The Geomagic Studio image processing software (12.0, Geomagic, USA) was then used to design the template outside the skin. One end (mounting sleeve) of the template was attached to the 2 external fixation pins, and the other end (guiding sleeve) was connected to the virtual cylindrical screw channel. The guiding sleeve was designed as 1-centered hole surrounded by 6 peripheral holes each with a uniform diameter of 2.5 mm, giving it a “plum blossom” like appearance (Figures 1(c) and 1(d)). The virtual template was then cut into 2 pieces and printed by the 3D equipment software (UnionTech, SLA-Lite 450 HD, China) using photosensitive resin material (accuracy, 0.1 mm) (Figure 1(e)). Finally, the components of the template were sterilized using low-temperature plasma for intraoperative use.

### 2.4. Surgical Procedure and Modified Screw Insertion Technique

Patients were placed in the supine position on the fluoroscopic operating table. The buttocks of the patients were elevated by 4-5 cm to avoid any obstruction by the table when inserting the IS screw. Meanwhile, a cylindrical pad was put behind the knees which flexed the hips and knees joint slightly and ensured the muscles and neurovascular bundle were not too tense.

**Figure 2:**
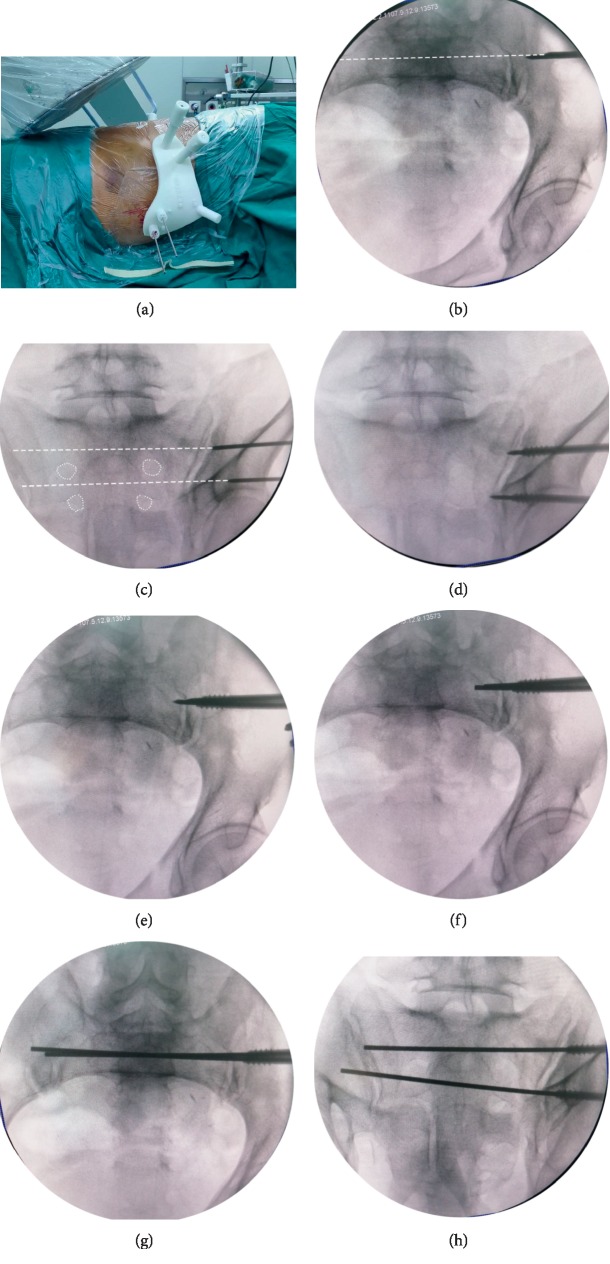
Photographs demonstrating the insertion of IS screws using “blunt end” KW technique. (a) The template was assembled intraoperatively, and a 2.5 mm KW was inserted in an antegrade fashion through the guiding hole. (b-c) Pelvic inlet and outlet fluoroscopy views were conducted to observe if the extension line (white dotted line) of KW was correctly positioned. (d-e) The KW was hammered until the tip reached the sacroiliac joint, and a 6.5 mm cannulated screw was inserted along the KW up to the sacroiliac joint. (f-h) The KW was pulled out and reversed such that its blunt end could be inserted through the hollow passage of the cannulated screw, and the blunt end was advanced inside the cancellous bone of sacrum until reaching the contralateral sacroiliac joint.

After satisfactory reduction, the template was firmly assembled to the marker pins (external fixation pins), and the depth of the template was determined according to the end of the mounting sleeves flush with the end of the marker pins (Figure 2(a)). Then, a 2.5 mm KW was inserted in an antegrade fashion through the guiding hole to pierce the skin to reach the bone surface. Next, without the need for the surgeon to maintain the hand-held KW, pelvic inlet and outlet fluoroscopy views were conducted to observe if the extension line of KW was correctly positioned (Figures 2(b) and 2(c)). If the entry point and direction of the KW were properly obtained, it was slowly hit with a hammer until the tip reached the sacroiliac joint (Figures 2(d) and 2(e)). The position of the KW was reconfirmed under fluoroscopy. Subsequently, the template was removed and a 6.5 mm cannulated screw with appropriate length was inserted along the KW up to the sacroiliac joint. After confirming that the screw was secure, the KW was pulled out and reversed such that its blunt end could be inserted through the hollow passage of the cannulated screw. Then, the blunt end was advanced inside the cancellous bone of sacrum until it reached the contralateral sacroiliac joint. Fluoroscopy inlet and outlet views were obtained to confirm the desired position of the wire (Figures 2(f)–2(h)), and then the residual part of the cannulated screw was screwed in. If a transiliac-transsacral screw was necessary, the wire could be inserted through the contralateral sacroiliac joint using a hollow electrical drill.

**Figure 3:**
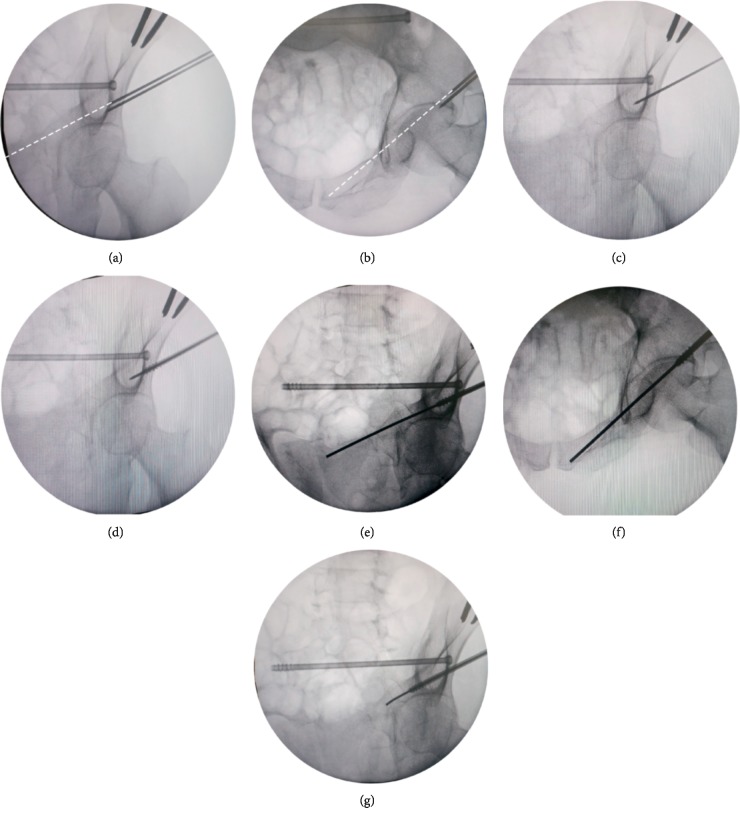
Photographs demonstrating the insertion of antegrade superior ramus screws using the “blunt end” KW technique. (a-b) The position of the inferior KW inserted through the centered hole of the guide tunnel was not ideal, and then a second KW was inserted through 1 of the peripheral holes of the “plum blossom” until an ideal position was obtained (white dotted line). (c) Obturator outlet view was taken to confirm the position was good after inserting the KW 1-2 cm into the bone. (d) The preselected cannulated screw was inserted along the wire. (e-f) The “blunt end” KW technique was used again to complete the insertion of the guide wire under the surveillance of intraoperative fluoroscopic pelvic inlet and obturator outlet views. (g) The “sea snake head” technique was used so that the curved tip of KW could pass through the narrow corridor or broken end of the fracture smoothly.

For the fixation of superior ramus fractures, the insertion of antegrade superior ramus screws is preferred as the template of the antegrade screw, providing more maneuverability, and it can be designed to be integrated with the template of IS screw to save materials. Similarly, the KW was inserted in an antegrade fashion through the centered hole of the guide tunnel, and it was ensured that the sharp end could easily touch the bone surface avoiding malposition of the KW caused by the soft tissue outside the iliac bone. Inlet and obturator outlet views were taken to observe the position of the wire. If the position was not ideal, one of the peripheral holes of the “plum blossom” was selected, and a second KW was inserted until an ideal position was obtained (Figures 3(a) and 3(b)). Unlike IS screw insertion, because the angulation between the longitudinal axis of the anterior column and the posterolateral surface of the iliac wing was so small (30°) [16], the tip of the KW could easily slip onto the bone surface, thus failing to enter the cortical bone from the predetermined optimal insertion point. Therefore, at the initial wire insertion, helped by an assistant, the surgeon should hold the wire steady with a gauze in one hand and, an electric drill in the other hand, rotating the KW at a high speed and slowly putting it forward at the same time until the wire was inserted 1-2 cm into the bone. The electrical drill was then been taken off and fluoroscopy views obtained to confirm the good position (Figure 3(c)). The template was also removed, and subsequently the preselected cannulated screw was inserted along the wire about 2 cm (Figure 3(d)). Again, superior ramus screw insertion could be accomplished successfully using the “blunt end” KW technique mentioned above (Figures 3(e) and 3(f)). In some cases, the bony corridor of superior ramus was narrow, or the blunt end of the KW was protruded from the fracture end. In these cases, the “sea snake head” technique could be used. Specifically, the blunt end of the wire was slightly bent by 5–10°, so that the KW could pass through the broken end of the fracture smoothly (Figure 3(g)).

In some cases with large superior ramus fracture displacements or combined pubic symphysis separation, the INFIX replaced the superior ramus screws to stabilize the anterior pelvic ring [17].

### 2.5. Postoperative Management and Assessment

First-generation cephalosporin antibiotics were used for 1–3 days after surgery, and oral administration of eliquis (apixaban, 2.5 mg/bid) was started the day after surgery. Patients were encouraged to reduce their time in bed less and perform active and passive exercises of the hip joints in the early stage of recovery. After the operation, X-rays of the pelvis were taken at anterior-posterior, inlet and outlet views, and a pelvic CT scan was performed. Patients were asked to carry out regular outpatient checkups. They were given partial weight-bearing with double crutches 2–4 weeks after surgery and asked to walk with single crutches 4–10 weeks after surgery and walk with full weight-bearing 10–12 weeks after surgery.

According to the results of the postoperative X-ray and CT scan, the fracture reduction was evaluated by using Matta's criteria [18]. A maximum distance of fracture displacement less than 4 mm is considered excellent, 4∼10 mm is good, 10∼20 mm is fair, and greater than 20 mm is poor. The Majeed score system [19] was used to assess pain (30 points), work (20 points), sitting (10 points), sexual intercourse (4 points), and standing (36 points). The recovery was divided into 4 grades: excellent 85, good 70–85, medium 55–69, and poor 55. Excellent and good rate = (excellent + good)/total cases.

## 3. Results

### 3.1. General Results

All 28 patients completed the operation successfully. The number of fluoroscopies was 28–60, with an average of 35, and the operation time was 60–150 min, with an average of 85.2 min. A total of 15 superior ramus screws (cannulated screws with a diameter of 6.5 mm) were placed in 28 patients, including 11 anterograde superior ramus screws and 4 retrograde superior ramus screws. INFIX was used to fix the anterior pelvic ring in 17 patients. A total of 19 IS screws (length 65–140 mm) were inserted in S1 and 28 IS screws (75–140 mm) in S2. The average time required for the placement of S1, S2, anterograde superior ramus, and retrograde superior ramus screws was 19.6, 19.0, 25.0, and 25.8 minutes, respectively. The average postoperative hospital stay was 5.8 days (range 3–12 days). More details are listed in Table 1. All patients were followed up for 12 to 24 months, with an average follow-up of 18 months. No complications such as loosening of the internal fixator or redisplacement of the fracture occurred during the follow-up period.

### 3.2. Imaging Evaluation and Functional Score of Fracture Reduction

Postoperative reduction was evaluated by Matta's criteria [18] as follows: excellent in 16 cases, good in 9 cases, and fair in 3 cases. The proportion of excellent and good cases was 89.3% (25/28). The Majeed pelvic fracture function score system [19] was used in the last follow-up to evaluate functional recovery as follows: excellent in 13 cases, good in 10 cases, fair in 4 cases, and poor in 1 case. The proportion of excellent and good cases was 82.1% (23/28).

### 3.3. Complications

All 28 patients completed the operation successfully, and no vascular injuries occurred during the operation. One patient was found to have S1 screw perforation but without any symptoms, and 4 patients showed lateral femoral cutaneous nerve injury symptoms. No other complications such as injury of the iliac vessels or pelvic organs occurred during the operation.

## 4. Discussion

Based on the current study, with the assistance of personalized 3D printed external template, the “blunt end” KW technique allows for successful insertion of percutaneous IS screws and superior ramus screws (antegrade or retrograde), resulting in satisfactory clinical outcomes for the treatment of unstable pelvic fractures. From the early days of open reduction and internal fixation surgeries using reconstruction plates to more recent closed reduction and external fixation surgeries using external pins, minimally invasive internal fixation using screws has been widely accepted today for the treatment of unstable pelvic fractures [1, 3, 20, 21]. Compared with traditional open reduction and plating fixation, percutaneous screw fixation has several advantages such as less trauma, less bleeding, and a more stable and rapid recovery of patients. It can also avoid the shortcomings of external fixators such as pin infection, loosening, obstruction of patients' daily activities, and nursing. Therefore, the minimally invasive screwing fixation has become the most popular and effective method for the treatment of unstable pelvic fractures [7, 22].

The corridor screw technique is the core of minimally invasive screw fixation for pelvic fractures. However, performing an optimal insertion of various pelvic corridor screws is full of difficulties and challenges, especially for the insertion of S1/S2 IS screws and superior ramus screws (antegrade or retrograde). The osseous channel of screw is narrow and surrounded by countless vessels and nerves; therefore, the percutaneous technique has a long learning curve and requires extensive experience. Conventionally, under the guidance of X-ray fluoroscopy, the occurrence of screw malpositioning and prolonged radiation exposure to both patients and medical staff is relatively high. In addition, even a slight screw malpositioning by less than 4° can lead to catastrophic neurovascular injuries [23]. For these reasons, many orthopedists have been discouraged from developing this technique. On the other hand, this has prompted many innovations in medical instruments and equipment, such as appearance of intraoperative CT, 3D navigation, and surgical robots [7, 10]. However, their high cost and the complexity of the setup have limited the widespread use of these equipments in most hospitals.

Recently, the use of 3D printing technology has been rapidly rising in the medical field. It has been reported that personalized internal templates based on 3D printing technology were used to assist the insertion of IS screws [12]. Although the internal template provides a beneficial guide for screw orientation resulting in accurate positioning of screws, it requires a 4-5 cm incision at the posterior iliac crest and the attached soft tissue should be dissected completely to provide a clear bone surface to match the template. The geometry of the iliac crest is inadequate to restrain the internal template, and it is difficult to determine accurate anatomical markers to match with the template intraoperatively, leading to high risk of slippage and malpositioning of the IS screw. In addition, soft tissue residues may also contribute to a mismatch between the template and bone surface.

Based on this, our team envisaged whether we could turn this abstract anatomical marker in vivo into a concrete marker in vitro [13]. Inspired by the external fixation pins inserted in the iliac crest, we took 2 external fixation pins as an ideal marker to design an external template, which could overcome the abovementioned shortcomings of the internal template. One end of the external template was firmly attached to 2 nonparallel cylindrical external fixation pins, and the other end was the guide channel to give a correct spatial orientation of the corridor screws of pelvis. In order to address possible deviations, the guide channel was designed in the shape of “plum blossom.” If it was found that the central channel of “plum blossom” presented with a little deviation, the surgeon could adjust and select 1 of the peripheral channels according to the intraoperative fluoroscopy.

However, an extra process of the external fixation pins insertion is a limitation of this technique before the definitive operation. This is of particular note in these patients who did not need emergent external fixation to stabilize the pelvis, a sufficient doctor-patient communication and informed consent was necessary. A delay in the definitive operation is another potential weakness of our technique because of the time needed to design and 3D-print the external template. The preoperative preparation included the emergent insertion of external fixation pins after admission and performance of pelvic CT scan, obtaining DICOM data for the design of the external template and finally printing and sterilizing the template for the definitive surgery. It should be noted however that the whole process is not overly complicated and can be completed within 3 days after the first attempt. The mean time from admission to definitive surgery in our study was 4.5 days, which was acceptable and had no influence on the quality of fracture reduction.

As previously mentioned, the external template provides an accurate orientation for guiding wire insertion. As such, how can we insert the guiding wire as safely as possible? During the insertion of the guiding wire (KW), it is difficult for the surgeon to ensure that the sharp end of the wire will not penetrate the cortical bone by relying solely on 2D intraoperative fluoroscopy. Moreover, it is easier for the sharp end to penetrate the cortical bone when there are anatomical variations or narrow corridors as seen in both IS and superior ramus screws. To overcome this shortcoming, the “blunt end” KW technique was adopted and instead of the sharp end, the blunt end of the KW was introduced into the osseous channel, which is similar to the insertion of the guiding wire used for intramedullary nail fixation [4]. During the process of inserting the guiding wire of intramedullary nails, the surgeon does not have so many concerns, partly due to the blunt end design of guiding wire. Therefore, for the insertion of pelvic corridor screws, introducing the KW by freehand hammering allows more sensitive palpation of the location of wire, which would make surgeons feel more secure [14]. Therefore, it is a very practical surgical technique to combine the “hammering technique” [24] with the “blunt end” KW technique, thus reducing the risk of penetration and neurovascular injury.

The “blunt end” KW technique could be used alone or in combination with template or navigation technology. All 28 patients with unstable pelvic fractures in our study were treated surgically by adopting this combined method. Unstable pelvic fractures mainly included Tile's classification [15] type B and C fractures. A type B fracture is a pelvic fracture with rotatory instability and vertical stability of the pelvic ring, so it is necessary to stabilize the anterior ring of the pelvis, sometimes at the same time as the posterior ring of the pelvis. A type C fracture shows rotatory instability associated with vertical instability of the pelvic ring, so the anterior and posterior rings of the pelvis must be stabilized simultaneously. In our study, combined with the assistance of a 3D printed external template, the “blunt end” KW technique provided an accurate and fast method to insert the percutaneous IS screws for stabilizing the posterior ring and superior ramus screws (antegrade or retrograde) for the anterior ring. All patients successfully completed the operation without any reports of vascular injury.

There are several points for attention while conducting this technology: (1) if the blunt end of the KW was found to penetrate through the broken end of the fracture, the “sea snake head” technique was used to bend the end of wire 5–10° in order to enter the medullary canal of the distal end of the fracture smoothly. (2) The external fixation pins (marker pins) on the iliac crest should not be disturbed before the operation as the design of the screw and template totally depends on the relative spatial position of the pins. In this way, we have manufactured a kind of “arch” aluminum alloy outer frame which covers the patient's abdomen to prevent bedding loosening the marker pins. (3) The 3D printed template needs low temperature plasma disinfection to prevent its deformation caused by high temperatures or liquid immersion, which can affect its accuracy. (4) During the operation, the template should be repeatedly assembled and disassembled. The operation process should be kept gentle as to avoid loosening of the external fixation pins or the KW, resulting in a decrease in accuracy.

In addition, this template based on external fixation pins still requires some technical improvements; for example, the current external fixation pins are made of stainless steel thus artifacts are found on the CT images. Therefore, an engineer needs to spend time removing the artifacts around the positioning pin, and this can affect the precision of the template. This leaves us with 1 question: how can we obtain a better material to manufacture the marker pins and thus eliminate, as much as possible, the CT image artifacts?

## 5. Conclusions

In short, the “blunt end” KW technique assisted by 3D printed external template is a safe and effective method for the placement of IS and superior ramus screws in unstable pelvic fractures. It provides an accurate and safe approach for the placement of pelvic corridor screws with a minimized surgical duration, less radiation exposure, and less surgical complications.

## Figures and Tables

**Table 1:** Demographic characteristics of 28 patients with unstable pelvic fracture.

Patient no.	Sex	Age (years)	Tile	Number of fluoroscopies	Operative time (min)	Operative time per screw (number of screws) (min)				Postoperative hospital stay (days)
						S1	S2	ASRS	RSRS	
1	M	54	C1	47	105	19 (1)	15 (1)	25 (2)	—	5
2	M	27	C1	40	90	17 (1)	18 (1)	—	32 (1)	4
3	M	30	C2	60	150	25 (2)	20 (2)	—	—	7
4	M	67	C1	33	85	17 (1)	15 (1)	—	—	12
5	M	61	C1	30	70	16 (1)	12 (1)	—	—	5
6	M	47	C2	32	80	—	17 (1)	20 (1)	—	7
7	F	51	B2	28	70	15 (1)	13 (1)	—	—	5
8	M	68	C2	40	90	15 (1)	15 (1)	18 (1)	24 (1)	6
9	M	54	B2	28	60	—	28 (1)	—	—	3
10	F	47	B1	32	75	18 (1)	14 (1)	—	—	4
11	F	55	B1	30	75	—	27 (1)	35 (1)	—	5
12	M	38	B1	43	100	30 (1)	20 (1)	—	20 (1)	4
13	F	24	C1	36	85	20 (1)	15 (1)	—	—	4
14	F	18	B2	42	100	17 (1)	17 (1)	22 (2)	—	6
15	F	44	C1	35	95	—	23 (1)	20 (1)	—	5
16	M	49	B2	30	80	—	27 (1)	—	—	4
17	M	35	B2	32	90	20 (1)	17 (1)	—	—	6
18	M	47	B1	34	80	—	23 (1)	28 (1)	—	6
19	M	38	B2	40	90	—	20 (1)	30 (2)	—	5
20	M	37	B1	28	75	—	24 (1)	—	—	7
21	M	47	B2	34	90	18 (1)	16 (1)	—	—	8
22	F	39	B2	30	70	—	22 (1)	—	—	5
23	M	57	B1	40	80	16 (1)	15 (1)	—	27 (1)	6
24	M	37	C1	36	85	20 (1)	16 (1)	—	—	8
25	F	52	C1	42	90	22 (1)	18 (1)	—	—	4
26	M	54	C2	32	80	18 (1)	20 (1)	—	—	7
27	M	55	B2	30	75	—	26 (1)	—	—	5
28	F	68	B2	28	70	25 (1)	—	—	—	9

M: male; F: female; ASRS: antegrade superior ramus screw; RSRS: retrograde superior ramus screw.

## Data Availability

The data used to support the findings of this study are available from the corresponding author upon request.
